# Class and classification: the London Word Blind Centre for Dyslexic children, 1962–1972

**DOI:** 10.1080/03054985.2020.1751099

**Published:** 2020-08-13

**Authors:** William Whyte

**Affiliations:** St John’s College, Oxford, UK

**Keywords:** Dyslexia, psychology, education, social class, psychiatry

## Abstract

The Word Blind Centre for Dyslexic Children opened in London in 1963. It was not only the first clinic established in Britain specifically to cater for children diagnosed with dyslexia. It was also intended to provide compelling evidence that a condition called dyslexia actually existed. The results of this work were published in Sandhaya Naidoo’s path-breaking study, *Specific Dyslexia*, which did exactly what its promoters had hoped it would, drawing on in-depth studies of 196 children to argue that dyslexia was indeed a distinct ‘constitutional disorder’. Using the archives produced by Naidoo and other sources, my article offers the first-ever account of this pioneering enterprise, exploring the reasons the Centre was set up, the way it worked, and the consequences of its work. In particular, it focuses on the rationale for Naidoo’s report, which only dealt with the experiences of middle-class boys. This choice is highly revealing, illuminating attitudes to reading, to class and gender, and to the competition for authority amongst the professionals who sought to explore all these issues. An intriguing case study in its own right, this also sets the scene for many of the themes that follow in this Special Issue.

On 12 April 1962 something like 350 people attended a conference on ‘Word-Blindness, or Specific Developmental Dyslexia’ in London.^1^ Convened by the Invalid Children’s Aid Association (ICAA) and hosted by the Medical College at St Bartholomew’s Hospital, the sheer scale of the enterprise surprised even its organisers, who had expected less than a third of that number to turn up, and found themselves turning away disappointed prospective participants who ‘almost literally jammed the doors’ in their enthusiasm to join in (McLeod 1966, p. 14). The speakers included such international luminaries as James Roswell Gallagher, Harvard professor and ‘acknowledged founder of adolescent medicine’ (Prescott et al., 1996, p. 2); Ingrid Riis-Vestergaard, from the pioneering Word-Blind Institute in Copenhagen; Suzanne de Séchelles, a leading French speech and language therapist; and Donald Shankweiler, *en route* from the US National Institute of Health to a long and distinguished career as an eminent psychologist at the University of Connecticut. The attendees were more insular in origin, but still a variegated group:

neurologists, paediatricians, Medical Officers of Health, psychiatrists, psychologists, Social Workers, various professors concerned with language and education, apart from school teachers and just some plain parents,

as the chairman of the ICAA, Dr Alfred White Franklin, observed at the start of the event (White Franklin, 1962, p. 4).

All looked set for a successful conference: one intended ‘to ventilate what is known and thought about the diagnosis and treatment of specific developmental dyslexia’ (White Franklin, 1962: n.p.). There was even a letter of support from Princess Margaret, patron of the ICAA. Yet, as one speaker recalled more than forty years later, the conference was in fact ‘a decidedly stormy one’ (Miles, 2006, p. 28). Far from providing a dispassionate discussion about the causes and cures of a ‘rare’, but serious condition, believed to ‘adversely affect the school progress, the attitude to learning, the general behaviour and the emotional development of the sufferers’ (White Franklin, 1962, n.p.), it proved a disappointment to its organisers, whose irritation is evident in the transcript of the event. Summing up the proceedings, Alfred White Franklin expressed his sadness that so little of value had emerged from the conference. Before the meeting, he observed, the Invalid Children’s Aid Association had imagined creating a centre in or near London where children could be assessed and treated and teachers trained to support them. Now, however, he had come to the conclusion that the ICAA was not the appropriate vehicle for achieving this goal. Indeed, White Franklin concluded, the ‘only positive thing’ to emerge was the proposal that the families of children with dyslexia should found a forum of their own: a ‘can’t read/can’t spell parents’ association’ (Naidoo, 1972, p. xiii).

Despite the organisers’ disappointment, both the decision to call a conference and the disputatious nature of the event itself are extremely revealing. They reveal the growing interest of a range of people in the problems some children were having with reading at the turn of the 1960s. They reveal the emotions that this phenomenon evoked – and the fact that it was not only parents who were strongly invested in the subject (for the parents, see Kirby, 2019a). Professionals, it seems, were just as engaged and equally likely to lose their temper as those with apparently more direct and personal connections to the problem. And this reveals another important issue: the striking range of experts who claimed expertise in this field, each with different specialities and varying degrees of authority. The role of the conference convenors is also worth considering. The decision to call a conference on dyslexia represented a change of direction for the Invalid Children’s Aid Association: a new focus that spoke of changing priorities and of the altered environment within which the ICAA worked. Above all, the conference was important because it did not simply end in acrimony. Notwithstanding the arguments, the organisers determined to do something about dyslexia, establishing a ‘Word-Blind Committee’ and then a Centre for Dyslexic Children.

This article is an attempt to explore that decision, the institution that was its consequence, and the context within which it operated. Although not the first study to engage with the subject, it does approach it in different ways, by remaining focused on the tensions that brought the Centre into existence, continued to underwrite its operation, and ultimately jeopardised its success (Beard, 2019; Kirby, 2019b).

Broadly understood, these tensions were two-fold, concerned with the interconnected issues of both class and classification. On the one hand, as the very title chosen suggested, there was a real ambiguity about what the Word Blind Centre for Dyslexic Children was intended to deal with. Was it Word Blindness (a relatively outdated term by the 1960s) or was it dyslexia (an increasingly popular, but highly contested concept)? On the other hand, there was an equally unresolved set of questions about whom this condition affected, how they were most effectively identified, and which professionals were best able to deal with them. Was this problem with reading confined to the intelligent or was it more widespread? Was it a neurological, a psychological, a social, or simply an educational deficit? The ICAA’s ambitious project never really resolved any of these tensions; indeed, it may have made them worse. Certainly, by 1972, as the Word Blind Centre for Dyslexic Children was wound up, its director’s attempts to find a resolution by highlighting the cases of a narrow class of children not only failed to address the doubts of critics but also unwittingly presented still more problems for those concerned with the subject.

The World Blind Centre thus stood at the intersection of several important themes in the history of dyslexia. It was a place where debates about divergent accounts of the phenomenon were played out: some medical, some psychological, some educational. It was an enterprise that illuminates the ways in which these debates were themselves framed by other issues. Discussions about literacy were closely connected to commonly accepted ideas about class. Matters of social and professional status also affected how competing professionals related both to one another and to the subjects of their research. Viewed in this light, the Word Blind Centre vividly illustrates the interrelationship of notions about class and classification, with arguments never just about dyslexia, but also how attitudes to the diagnosis and treatment of dyslexia were shaped by wider trends. A fundamental problem at the time, it is one that has continued to dog the discipline to this day.

## Origins

The conference of 1962 and the Centre which it inspired were the products of a particular milieu and a distinctive moment in time. Not least, they reflected the ambiguous impact of the post-war welfare state on voluntary bodies and charities which now sought a new purpose and reason for being (Beveridge & Mold, 2011). This was certainly true of the Invalid Children’s Aid Association, set up in 1888 as an offshoot of the Charity Organisation Society, which, as Jane Lewis has noted, ‘occupied a dominant position in terms of influencing opinion on welfare issues’ in the late-nineteenth century, ‘particularly in terms of advocating a large role for charity and a small one for the state’ (Lewis, 1995, p. 5). The ICAA applied the practices of its parent organisation to ‘seriously invalided children’: visiting, befriending, and supporting them to become useful citizens. But the creation of the National Health Service in 1948 seemed to make much of this work redundant, and so the Association turned to other activities, moving, as the director observed in 1961, ‘from its primary interest in physical environment to the problems that arise because a child cannot adjust to school life’ (*Lancet*, 1961, p. 955). Increasingly, as the Association’s historian has shown, this meant ‘helping children with communication difficulties’ (Rackham, 1978, p. 24).

The ICAA’s interest in children’s communication difficulties undoubtedly reflected its need to find a purpose. But this choice of focus was by no means arbitrary or ill-informed; rather, it reflected a growing recognition of a well-defined problem. The post-war settlement had provided a network of support for children that was not confined to the familiar triad of ‘state orange juice, milk, and school dinners’ explored so fully and so fascinatingly in previous studies (Thomson, 2013, p. 81). It set up near universal provision of School Medical Services and made ambitious plans to establish a widespread system of Child Guidance Clinics. It had also driven improvements in mainstream and what was increasingly known as ‘special’ education, with the result that literacy rates had risen. In 1961, it was recorded that the percentage of ‘illiterate’ 15 year-olds had fallen from 1.0% to 0.0% since 1948 and the proportion of ‘semi-literates’ had similarly declined from 5.0% to 2.0% (Vernon, 1966, p. 148).

Notwithstanding these encouraging headline figures, there remained a small residuum of students who never learnt to read or struggled with language more generally. The number of children sent to speech therapists in England and Wales rose from 16,000 in 1947 to nearly 50,000 in 1957. It would rise again to almost 70,000 in 1967 (Harris, 1995, p. 189). Others were referred to Child Guidance Centres, which saw an equally exponential rise in demand – and similarly struggled to provide remediation for these problems. It was, after all, just such a difficult case at his local Child Guidance Clinic in 1949 that first interested Tim Miles in the subject – and helped inspire his career as a leading expert on dyslexia (Evans, 2020; Miles, 2006). The Ministry of Education’s 1946 report on *Special Educational Treatment* noted that little was known about how to deal with these children and no special schools existed to help them. ‘Further research and experiment’, it went on, ‘may cast light on an obscure condition and suggest more satisfactory means of educating aphasic children’ (Ministry of Education, 1946, p. 31). But this research had been piecemeal, and by the middle of the 1950s, it remained the case that specialist provision was patchy at best and much more often simply absent. There was a lack of Child Guidance Clinics and a dearth of professionals to work in them. By the mid-1950s only 32 of the 96 Local Education Authorities had established Clinics and fewer than half of the 640 specialists they required to function fully had been trained and recruited (Hurt, 1988, p. 178).

The ICAA had thus chosen a field in which it really could make a difference. There was a demonstrable, if intractable, problem and a growing awareness that state provision was not adequately addressing it. Moreover, it was a problem that had become increasingly interesting to scientists, especially psychologists, who were experiencing what Bonnie Evans has termed a ‘linguistic turn’, as more and more of them devoted attention to language and its disorders (Evans, 2017). In 1958 the Association founded only the second school in the country for children with serious speech and language problems. In 1962, it opened another small school for children ‘whose almost total inability to communicate led to severe behaviour disorders’ (Rackham, 1978, p. 26). In 1964, it would convene another conference on *Children with communication problems* (White Franklin, 1965). Word-Blindness, or Dyslexia, was a natural subset of this interest, providing a good opportunity for the ICAA to contribute to an issue that the welfare state had raised but not solved.

Yet if this field was uncharted and apparently promising, it was also potentially hazardous. In leaving behind the Association’s traditional expertise in medicine and social work, it ventured into disputed territory. The world of child development in general – and that of Child Guidance Clinics in particular – was notoriously fractious and divided into hostile camps. Psychologists and psychiatrists differed on diagnosis and on treatment. At a major conference in 1951, it was not only noted that their interactions were characterised by ‘tension, and at times bitterness’, but also that they were often ‘remarkably rude to one another’ (Stewart, 2013, pp. 149–50). There was likewise a ‘long-standing professional antagonism between doctors and psychologists’, who similarly doubted the professional competence of each other (Thom, 1992, p. 202). Teachers, too, competed for authority; as did educational psychologists, most of whom began their careers as teachers. There were signs of this at the ICAA’s first meeting on dyslexia in 1961, which opened with a talk by the influential neurologist Macdonald Critchley, who argued for a neurological concept of ‘developmental dyslexia’. The event, however, closed with testimony from ‘a school-teacher who put the case for those who regard dyslexia as nothing more than a severe form of slow reading associated with emotional disturbance’ (*Lancet*, 1961, p. 955). This was an augury of things to come.

## Disputes

Longstanding divisions within child development were likewise made manifest at the ICAA’s 1962 conference. As White Franklin observed, ‘The majority of those’ who opposed the idea of dyslexia ‘were educational psychologists’ (White Franklin, 1962, n.p.). Their case was put, and with no little belligerence, by J. C. Daniels of the Institute of Education at the University of Nottingham: an educational psychologist with a decade’s experience of research on reading and a successful reading scheme of his own to promote (Daniels & Diack, 1954). In a forthright paper and in discussion, during which ‘Dr Daniels was very forceful and … used a blackboard to illustrate his points’ (White Franklin, 1962, p. 111), he argued strongly against the concept of dyslexia, maintaining that ‘practically every child of IQ over 70 can effectively be taught to read’, and urged that the term ‘word blindness’ be rejected altogether (Daniels, 1962, p. 88). A proponent of ‘the phonic word method of teaching reading’, he preferred to talk of ‘tone deafness’, and expressed total confidence that proper training could overcome virtually all problems with literacy (Daniels, 1957). He was not alone. Many other educational psychologists present shared this scepticism – and were unafraid to voice it. One observed that the representative body of the profession, the English Division of Professional Psychologists, had spent a week debating dyslexia the year before ‘and were quite unable to identify it’ (White Franklin, 1962, p. 129).

By contrast, the great advocates of the concept were predominantly drawn from a medical background: whether Macdonald Critchley, whose guidance had shaped the whole event, or the less famous but almost equally eminent neurologist Cecil Charles Worster-Drought. Those psychologists in favour also tended to be linked to hospitals, rather than schools. They included Margaret Reinhold and Oliver Zangwill, of the National Hospital for Nervous Diseases, and Maisie Holt, who was already running a clinic for dyslexic children at St Bartholomew’s. All these people shared a sense that the problem was medical rather more than educational. Zangwill had argued that dyslexia was linked to handedness and thus likely to be neurological (Zangwill, 1960). Building on this, Margaret Reinhold had recently published a paper similarly suggesting that ‘congenital dyslexia’ was ‘due to an inherited defect of function of the brain’ (Gooddy & Reinhold, 1961, p. 242).

These two tribes of specialists might have been speaking different languages; indeed, it became clear they were. A third dialect was added by the few psychiatrists present. One of these failed to turn discussion towards a subject that her home institution, the Tavistock Clinic, had made its own by asking ‘whether perhaps we had investigated … emotional difficulties enough’ (White Franklin, 1962, p. 81). No one answered. Another sought to bring peace – but only at the expense of striking professional abnegation, disapproving of any idea that

We, the psychiatrist, the psychologist, the psychiatric social worker, can presume to disagree with the neurologist, who has far better grounds than us for agreeing that there is such a concept of specific developmental dyslexia (White Franklin, 1962, pp. 130–31).

Again, no-one responded favourably to her suggestion. Despite the urgings of the psychologist Patrick Meredith, who made ‘an appeal not to let the children become the shuttlecock of a professional controversy’ (White Franklin, 1962, p. 138), the conference revealed profound differences between these rival specialisms.

Such differences were the consequence of very different experiences. For the neurologists and for many psychologists, reading disorders were a relatively new area of serious interest, with dyslexia first described by their predecessors at the turn of the twentieth century and then neglected in subsequent decades. As Macdonald Critchley observed in 1964, this history resulted in the ‘unexpected and unfortunate effect’ of leaving the subject to the educational psychologists, whose interests and expectations were very different and whose limitations had tended to ‘obscure the issue at times’ (Critchley, 1964, p. vii). For the educational psychologists, by contrast, literacy was a long-standing interest, and the influence of doctors and experimental psychologists had proved far from helpful in the past. They were outraged at the assumption that this field had been neglected, indignantly observing that they had been working on the problem for decades (see especially A. G. Walbridge in White Franklin, 1962, p. 106). They were also infuriated by the presumption of these medical rivals, who lacked their experience of actually teaching children. At the conference, the disputatious Dr Daniels argued that the abstractions of the medics would be ‘a ball and chain’ for teachers, who would be discouraged from helping any child believed to be congenitally incapable of reading (White Franklin, 1962, p. 135). Later still, other experts would note the resistance of educationalists to the way in which ‘the medical specialists ‘invaded’ an educational domain’ (Reid, 1968, p. 127).

All this bore out the rueful reflections of the psychologist Tim Miles at the Second International Reading Symposium of 1964. There he noted the ongoing division of opinion between ‘doctors, neurologists and neurologically orientated psychologists’, on the one hand, and ‘educational psychologists’, on the other. He also deprecated the ‘danger of believing that one’s own group … comprises the people who are really qualified to judge’ and the temptation ‘to attack those with whom one disagrees by disputing their qualification’ (Miles, 1967, p. 244). Such, indeed, had proved – and continued to prove – to be the case.

This division was long-standing because it was more than merely academic. It was both professional and personal. It was to do with class and with status. As Adrian Wooldridge has argued, educational psychology ‘only managed to win a marginal position in English professional life’ in the mid-twentieth century and educational psychologists ‘failed to command the rewards which they felt were commensurate with their intellectual ability and professional dedication’ (Wooldridge, 1994. pp. 153, 216). They tended to come from more working- and lower-middle class backgrounds than their medical rivals. They were less likely to have attended public school or the ancient universities. Most spent time as teachers before they became academics. Almost none was elected to the fellowship of either the British Academy or Royal Society – and there were for many years very few Chairs in psychology in Britain to which they might be promoted.

The distinction between these marginal men and the medical establishment is vividly illustrated in the battle between Alfred White Franklin and J. C. Daniels at the 1962 conference. Daniels was ‘the son of a miner and native of a small North Staffordshire village’ (Daniels & Diack, 1954). He had trained as a teacher and then taught in schools around Manchester before taking up a post at the Nottingham Institute of Education, a somewhat semi-detached part of what was then University College, Nottingham (Daniels, 1962, p. 79; Gower, 2014). White Franklin, by contrast, was a doctor, from a distinguished family of doctors. Educated at a public school in Surrey, at Cambridge, and then at St Bartholomew’s Hospital, he was a member of the exclusive and intellectually elitist London club, the Athenaeum, and a figure of such obvious and unimpeachable respectability that he was known as ‘the Bishop’ by friends (*Times*, 4 October 1984, p. 16).

## Foundations

The London Word Blind Centre was established with this row still raging and these tensions still raw. It is no exaggeration to say that it was set up in the hope of ending such disputes for good. Yet, in reality, it could do no such thing. The complicated conflicts over class that were confirmed by the 1962 conference did not dissipate; class would indeed become an ever more important issue in this field. Disputes about professional competence were likewise not easily resolved. Above all – and fundamentally linked to these underlying problems – there remained the question of what, if anything, dyslexia amounted to. As a result of these disputes, the Centre would be intended not merely to help those with this condition and offer examples of best practice for their teachers and parents. It would also be expected to demonstrate conclusively that the problem existed. This would prove to be the biggest challenge – and one that it struggled with throughout its existence.

Although it remained open for only a few years, from 1964 to 1972, previous studies of the Word Blind Centre have quite rightly pointed to its success. They have stressed its status as a pioneering institution (Beard, 2019). They have also noted how important it proved to be in stimulating other efforts. ‘As a nexus of interested scholars and advocates’, observes Philip Kirby, ‘the centre laid the foundation for the institutionalization of dyslexia in a series of later organizations, including research centres and advocacy organisations such as the British Dyslexia Association’ (Kirby, 2019b). It was a place that attracted hundreds of children for assessment and teaching – and a resource that genuinely improved their educational outcomes. A small study of its graduates conducted some years after it closed concluded that the Centre had effected a substantial improvement in their literacy (Bruce, 1983, p. 20).

Previous writers have also noted that all this achievement sat in stark contrast to the environment in which the Centre operated. Initially founded in the basement of the ICAA’s offices in Kensington, where teachers worked with children ‘whilst seeing disembodied feet’ walking along the pavement outside (Beard, 2019, p. 121), the Word Blind Centre acquired its own premises in 1965, moving to ‘a small school building on the point of being demolished in Coram’s Fields’, loaned to it by the Institute of Child Health at Great Ormond Street Hospital (White Franklin in Naidoo, 1972, p. xiv). It was a symbolically rich location: one that linked this work to a long tradition of concern for children in that place. But it was a far from prepossessing setting for the Centre: a redundant ‘lavatory block’ (Beard, 2019, p. 130) that would be supplemented by temporary buildings – ‘two caravans’, in the words of one supporter – as demand for services grew (Miles, 2006, p. 30).

Yet if the work of other scholars means that we now possess a good understanding of both the Centre’s importance and its rather ramshackle foundations, we have a less developed sense of just how ambiguous – even precarious – it proved intellectually. It is not simply that it was the product of an almighty row; nor indeed that the meetings of the Word Blind Committee which oversaw it were similarly stormy, characterised by one participant as ‘tempestuous affairs’ (Beard, 2019, p. 120). It is, still more, essential to recognise how remarkably unprepared the Centre was to solve the key conundrum which had prompted its creation: the definition and defence of dyslexia itself.

The founding director was a 39-year-old psychologist, Alexander Bannatyne. Originally from New Zealand, where he had studied education and philosophy, but with a doctorate from London’s Institute of Psychiatry, he was in many respects the ideal compromise candidate: a specialist in educational psychology and neuropsychology, with an interest in child development and in both handedness and the dominance of spheres in the brain (Jones, 2007, p. 237). He was, however, no expert on literacy, freely admitting in 1966 that

Although I have been involved in education and psychology for some twenty years, and in remedial education for some of that period, it is only in the last three or four years I have developed an intense interest in reading disabilities in children of normal intelligence. (Bannatyne, 1966, p. 20)

The Centre was thus, in his words, ‘the first major opportunity I had to pursue my studies of reading disability’ (Bannatyne, 1971, p. xi).

Perhaps, as a result, his initial conclusions are best described as inconclusive. Based on the study of 150 children who had attended the Word Blind Centre in its first year of operation, Bannatyne offered not one but ‘several ‘species’ of dyslexia’: an aetiology that was seemingly designed to satisfy each of the possible approaches previously presented to the subject. The first drew on the dominant model of contemporary psychiatric thought. ‘Emotional Dyslexia’ had as its primary cause ‘a poor communicative relationship between mother and child’. It was, in other words, the model preferred by the single, disregarded psychiatrist at the 1962 conference. The second species was more closely linked to recent developments in psychology, with ‘Neurological Dysfunction Dyslexia’ defined as the result of ‘some *abnormal* qualitative difference of the brain’. The third was ‘Genetic Dyslexia’, which Bannatyne described as ‘the most difficult, most controversial, and I would venture to say, the most homogenous species of dyslexia’: a problem probably born of difficulties with auditory sequencing, auditory discrimination, and ‘associating auditory symbols with sequences of visual symbols’. The fourth and final species was one much in line with the arguments made by many educational psychologists. ‘Social-Cultural-Educational Dyslexia’ affected those children who had ‘not received enough direct or indirect training to learn any given linguistic processes’ (Bannatyne, 1966).

The limitations of this taxonomy were made painfully apparent in Bannatyne’s admission that these categories were not mutually exclusive. In any event, he would leave the Centre for a career in the United States in 1966: first at the University of Illinois and then at the Bannatyne Children’s Learning Centre in Miami, Florida (Bannatyne, 1974). But if he had not succeeded in defining a single species of dyslexia, the approach he took would nevertheless help to shape the future work of the Word Blind Centre. His successor as director was another neurologically informed educational psychologist. Sandhya Naidoo had a background in teaching and had written a recent master’s thesis on ambidextrous children with Oliver Zangwill, who had, of course, advanced the claim that dyslexia was likely to be neurological in origin. Naidoo’s job, it became clear, was to flesh out Bannatyne’s third type of dyslexia: ‘Genetic’, or what he also termed, ‘Specific Developmental Dyslexia’. The results of this labour would be published as *Specific Dyslexia* in 1972 and form the most lasting legacy of the Word Blind Centre’s research.

*Specific Dyslexia* is based on an investigation of 98 children aged between eight and nearly 13 who were seen at the Centre between January 1967 and March 1969. Far more than this were assessed in that period, but these subjects were chosen with care because they were understood to be most likely to reveal the existence of a congenital reading problem. ‘For the purpose of selecting children for this investigation’, Naidoo explained, ‘specific dyslexia is defined as a condition causing difficulty in learning to read and spell in physically normal intelligent children in spite of continuous schooling and in the absence of severe emotional disturbance’ (Naidoo, 1972, p. 24). They were, in other words, members of Bannatyne’s third species, and their problems were not considered likely to result from the ‘Emotional Dyslexia’, ‘Neurological Dysfunction Dyslexia’, or ‘Social-Cultural-Educational Dyslexia’, which he had characterised as the other prevailing types of the condition. To that end, all these children had to pass a ‘Full Scale IQ’ test, before being assessed as ‘Physically and grossly neurologically normal’. They could not have experienced a major absence from school, nor any more than three changes of school. They also had to display ‘No evidence of severe emotional disturbance on the psychologist’s examination of the child and interview with parents’ (Naidoo, 1972, p. 27). In order to offer some point of comparison, the Centre recruited another 98 children who passed all these tests, but did not have any problems in reading or spelling, and thus constituted a control group.

Intended, in White Franklin’s words, to be a ‘cool study in an area where great emotion is generated’ (in Naidoo, 1972, p. xv), the report advertised its strict scientific approach from the start. Even the illustration on the front cover was taken from a computerised cluster analysis used in the research (Beard, 2019, p. 146). Keen to distinguish her methods from Bannatyne’s rather impressionistic practices, Naidoo rejected his criteria for selection and subjected her subjects to a battery of tests. The psychologist alone was expected to administer

The Weschler Intelligence Scale for Children, Neal’s [sic] Analysis of Reading Ability, Schonell’s Graded Word Reading Test, Schonell’s Spelling Test, Wepman’s Test of Auditory Discrimination, Renfrew’s Articulation Attainment Test, a test of Phoneme Blending (Sound Blending), Benton’s Right/Left Discrimination Test, Stott’s Test of Motor Proficiency or a shortened form of the Oseretsky Test of Motor Ability, Benton’s Visual Retention Tests, two tests of Finger Differentiation and tests of hand, eye and foot preference. (Naidoo, 1972, p. 35)

The results of these tests were combined with school reports, which included ‘details of school attendance, parental interest and an estimation of intelligence’, and information obtained from parents, which included ‘details of developmental history, illnesses, behavioural problems, mother/child separations, and the presence of reading, spelling and speech difficulty and of left-handedness in the child’s immediate family’ (Naidoo, 1972, p. 35). Out of all this – and still other investigations – Naidoo and her team were able to develop an extraordinarily deep understanding of each individual and a complex sense of the bigger picture.^2^ The report concluded that Bannatyne’s species were unhelpfully stark. ‘Specific dyslexia’ existed, but was best understood as a continuum: a ‘constitutionally determined’ condition that was caused by ‘complex rather than single aetiological factors’ (Naidoo, 1972, p. 114).

In many respects more striking than this particular conclusion, however, was the more general question of inclusion within the study itself. All of the children it was based on were boys and almost all of them were from the middle – indeed, the upper-middle – class. In fact, no less than 40% of the control group was recruited from two upmarket London Prep Schools: Eaton House in Belgravia and the Hall School in Hampstead. The children with dyslexia were significantly smarter still. All told, indeed, 155 of the 196 boys tested came from social classes I and II (’Upper and Middle Class’). A single boy in the control group was the sole representative of social class V (‘Unskilled Working Class’). This bias to some extent reflected the sorts of children sent to the Centre. The ratio of boys to girls seen there was 5:1 and this meant that there was simply not a statistically significant enough pool of female candidates to test. It was also true that the Word Blind Centre was particularly attractive to the middle classes, who could afford the fees and the cost of travel, and who were better placed to obtain information about its work (Kirby, 2019a).

But there was more to it than that. In the first place, those overseeing the research were, in the main, more familiar and probably more comfortable with middle- and upper-class circles than the lives of other social groups. White Franklin’s origins were evidently, unabashedly elitist. Naidoo’s children were themselves at private school. With the patronage of Princess Margaret – a ‘very active President of the ICAA’ – and with the support of several society figures, the Centre itself was an upmarket endeavour (Bannatyne, 1971, p. xi). It is consequently unsurprising that the four ‘Histories’ of dyslexic children used to open Naidoo’s report focused particularly on the problems faced by the middle-class. One was of ‘Peter’, the product of ‘an ordinary, hardworking and united’ family; the others – ‘Martin’, ‘Margaret, Simon, and Donald’ – were all from professional backgrounds, and the text stressed the distress that their difficulties caused them and their ‘highly educated’ parents. ‘Margaret’, for instance, attended ‘a small private school of high academic standard’. ‘Martin’ was ‘clearly of University calibre and would like to go to University one day’ – and this at a time in which only the most privileged 8% of teenagers could aspire to any form of higher education. ‘Is this a minor problem?’ Naidoo asked. ‘“Minor” is a relative term. Compared with those who can barely read, Martin might almost be said to have no problem. But to Martin and his parents, this is little comfort’. It was with ‘Martin and the many like him’ that the report was chiefly concerned (Naidoo, 1972, p. 2, 4, 5).

Secondly, and still more importantly, it is evident that these implicit assumptions were translated into research methodologies. The middle-class child – and, above all else, the middle-class boy – was regarded simply as normative. As Carolyn Steedman has noted, there was a widespread acceptance of the idea that the offspring of the professional classes were ‘just ordinary children’. There was an equal tendency to see the children of the working class as not ‘normal’; indeed to regard them as inherently deficient – especially when it came to their use of language (Steedman, 1985, p. 150). The belief that ‘intellectually retarded children without an organic brain disorder never, or practically never, come from middle-class families’ was so widespread that it was repeated by contemporaries even when the evidence, in fact, suggested quite the contrary (Yule & Rutter, 1970, p. 58).^3^

The process of selection for this study was biased in favour of the middle class as a result. The criteria for inclusion prioritised performance in IQ tests, yet it was well understood at the time that middle-class children invariably outperformed their less privileged contemporaries (Douglas, 1964). The researchers also found that ‘Among those excluded on the grounds of emotional disturbance, the proportion of boys from State schools was greater than those from independent schools’ (Naidoo, 1972, p. 28). As the existence and extent of ‘emotional disturbance’ was determined in no small part by the subjective reports drawn up by these schools, there were undeniably class-based elements even to this apparently objective judgement.

## Responses

Both the conclusions presented and the assumptions made by *Specific Dyslexia* mirrored those of other workers in the field. At a series of lectures organised by the ICAA, one of the teachers at the Word Blind Centre observed quite frankly that ‘Working-class parents tend to be less concerned about academic success’ than their middle-class counterparts. ‘When the parents are ambitious the child has a greater sense of failure’ (Gill C. Cotterell, in White Franklin & Naidoo, 1970, p. 25). As Philip Kirby has shown, the support groups set up in this period were overwhelmingly middle class, and the schools established tended to cater to a fairly well-heeled social set (Kirby, 2019a). The parents who had pushed for change at the 1962 conference were themselves middle class. They included a journalist and a canon of St Albans. Their ambitions – ‘be it the 11-plus, be it five O-levels, be it two A-levels’ – were those of the middle class too (White Franklin, 1962, p. 111).

In other respects, however, Naidoo’s report was unfortunately framed – and still more unfortunately timed. Even before it was published, other experts cast doubts on its approach. The post-war period saw all those involved in the ‘psy-sciences’ invest huge energy and place enormous faith in the possibilities inherent in statistical analysis (Hayward, 2011). The result was a proliferation of tests and an expansion in the scale of studies, with huge cohort analyses undertaken. One such was the Isle of Wight study discussed by Maughan et al. elsewhere in this Special Issue. As early as 1968, its directors dismissed the likely future findings of the Word Blind Centre on the grounds that they could never be as representative as a larger-scale project: ‘The type of problems seen at one clinic naturally reflect the biases influencing referral to that clinic and the kind of services that it provides’. Only ‘epidemiological investigations of *total* child populations’, concluded Michael Rutter and his collaborators, could provide real answers to the question of whether dyslexia existed or not (Rutter et al., 1968, p. 280).

Allied to this specific problem were other, wider issues. In the first place, there was the growing strength, confidence, and numbers of the educational psychologists. This period witnessed a massive increase in the size and scale of educational studies in the United Kingdom, with the number of trainee teachers in Colleges of Education rising from 48,000 in 1960 to 95,000 eight years later. These were better qualified people and they were being taught by more confident and ambitious academics (Rowland & Hatch, 2007, p. 67). At the same time, there was a discernible rise in the ambitions and the attractiveness of the social sciences more generally, with sociology and psychology becoming seen as quintessentially modern subjects (Grimley, 2019). The result, as J. B. Thomas has shown, was an era of unprecedented expansion in the field of educational psychology. Twenty-one Chairs in psychology were created and psychologists occupied many of the Chairs in education. Publications in educational psychology grew at a faster rate than those in any cognate field (Thomas, 2007). This new breed of educational psychologist was not to be intimidated by the claims of medical men – no matter how socially well-connected they were. Moreover, the educational psychologists were becoming recognised as the real experts on the subject. Tellingly, when the government commissioned a report on *Children with Specific Reading Difficulties* in 1972, it relied on the guidance of Jack Tizard, a psychologist based at London’s Institute of Education, and explicitly rejected the arguments made ‘by certain neurologists’. The Tizard report concluded that ‘we are highly sceptical of the view that a syndrome of ‘developmental dyslexia’ with a specific underlying cause and specific symptoms has been identified’ (Tizard, 1972, p. 1, 3).

Still more fundamentally, the assumptions about class that underwrote Naidoo’s report on *Specific Dyslexia* proved to be problematic. In part because of the rise in importance of the social sciences, the 1960s also saw what has become known as the ‘rediscovery of poverty’: a process encapsulated in the foundation of the Child Poverty Action Group in 1965 (Lowe, 1995). It was, of course, possible to be concerned about middle-class children’s literacy and working-class children’s poverty – and, indeed, charities like the City Parochial Foundation funded work on the latter at the same time that it committed £10,000 to the Word Blind Centre (Davis, 2019). But in the competition for attention and, still more, in the fight for resources, the association between dyslexia and the middle classes was unhelpful – and would turn out to be damaging, with many coming to believe that the term was simply used as an excuse for school failure by privileged children (Kirby, 2019b).

## Conclusion

The London Word Bind Centre for Dyslexia Children achieved much in its short existence. It formed a focus for further work and a seedbed for other organisations. It helped those children who attended it. The research it supported was widely read and would inspire other researchers, as well as reassuring those already committed to the concept of dyslexia that it was a useful term. What the Centre could not do – and could hardly be expected to do – was to overcome the many stark divisions and sharp disagreements over dyslexia. Indeed, its methods and its conclusions may even have entrenched them. Certainly, the link between class and classification that was made plain between 1962 and 1972 would continue to haunt the study of dyslexia. It haunts it to this day.

## Notes

1. The only book-length study of the Centre insists that this took place in 1961, but this is a confusion with an earlier meeting. See Beard, 2019.

2. The individual files produced by this exercise are now held by the History of Dyslexia Archive at St John’s College, Oxford, and will be the subject of a future article.

3. To demonstrate this general trend, they cite (Stein & Susser, 1960), which does, in fact, conclude quite the reverse.

## References

[cit0001] Bannatyne, A. D. (1966). The aetiology of dyslexia. *The Slow Learning Child*, 13(1), 20–34. 10.1080/0156655660130104

[cit0002] Bannatyne, A. D. (1971). *Language, reading, and learning disabilities: Psychology, neuropsychology, diagnosis and remediation*. Charles C. Thomas.

[cit0003] Bannatyne, A. D. (1974). Reading: An auditory-vocal process. *Bulletin of the Orton Society*, 24(1), 87–102. 10.1007/BF02653533

[cit0004] Beard, J. (2019). *From Percy to Peter: A history of Dyslexia*. Waterside Press.

[cit0005] Beveridge, V., & Mold, A. (2011). Professionalism, new social movements and voluntary action in the 1960s and 1970s. In M. Hilton & J. McKay (Eds.), *The ages of voluntarism: How we got to the big society* (pp. 114–134). Oxford University Press.

[cit0006] Bruce, D. (1983). Coping with dyslexia. *Cambridge Journal of Education*, 13(3), 16–22. 10.1080/0305764830130303

[cit0007] Critchley, M. (1964). *Developmental Dyslexia*. William Heinemann.

[cit0008] Daniels, J. C. (1957), 'The phonic word method of teaching reading', The Slow Reading Child 4:1, 6–14.

[cit0009] Daniels, J. C. (1962). Research on streaming in primary school. *Forum*, 4(3), 79–84.

[cit0010] Daniels, J. C., & Diack, H. (1954). *Learning to read: An outline of a new teaching method*. News Chronicle.

[cit0011] Davis, J. (2019). Reshaping the welfare state? Voluntary action and community in London, 1960–75. In L. Goldman (Ed.), *Welfare and social reform in Britain since 1870: Essays in honour of José Harris* (pp. 197–212). Oxford University Press.

[cit0012] Douglas, J. W. B. (1964). *The home and the school: A study of ability and attainment in the primary school*. Macgibbon & Kee.

[cit0013] Evans, B. (2017). *The metamorphosis of autism: A history of child development in Britain*. Manchester University Press. 28654228

[cit0014] Evans, R. J. W. (2020). “a pioneer in context: t r miles and the bangor dyslexia unit”. Oxford Review of Education [this issue]

[cit0015] Gooddy, W., & Reinhold, M. (1961). Congenital dyslexia and asymmetry of cerebral function. *Brain*, 84(2), 231–242. 10.1093/brain/84.2.231 13706997

[cit0016] Gower, R. (2014). *A short history of the school of education at the University of Nottingham*. University of Nottingham.

[cit0017] Grimley, M. (2019). You got an ology? The backlash against sociology in Britain, *ca*. 1945–90. In L. Goldman (Ed.), *Welfare and social reform in Britain since 1870: Essays in honour of José Harris* (pp. 178–195). Oxford University Press.

[cit0018] Harris, B. (1995). *The health of the schoolchild: A history of the school medical service in England and Wales*. Open University Press.

[cit0019] Hayward, R. (2011). Medicine and the mind. In M. Jackson (Ed.), *The oxford handbook of the history of medicine*. Oxford University Press, 524-42.

[cit0020] Hurt, J. S. (1988). *Outside the mainstream: A history of special education*. B. T. Batsford.

[cit0021] Jones, E. (2007). Bannatyne, Alexander D.. In C. C. Reynolds & E. Fletcher-Janzen (Eds.), *Encyclopedia of special education* (Vol. 1, pp. 237). Wiley.

[cit0022] Kirby, P. (2019a). Worried Mothers? Gender, class, and the origins of the “dyslexia myth”. *Oral History*, 47(1), 92–104.

[cit0023] Kirby, P. (2019b). Literacy, advocacy and agency: The campaign for political recognition of dyslexia in Britain (1962–1997). *Social History of Medicine*. *Lancet* (1961), 277:7183, 955). https://academic.oup.com/shm/article/doi/10.1093/shm/hkz030/5423863 10.1093/shm/hkz030 PMC7805790 33469410

[cit0024] Lewis, J. (1995). Family provision of health and welfare in the mixed economy of care in the late-nineteenth and early twentieth century. *Social History of Medicine*, 8(1), 1–16. 10.1093/shm/8.1.1 11639612

[cit0025] Lowe, R. (1995). ‘The rediscovery of poverty and the creation of the child poverty action group, 1962–68ʹ. *Contemporary Record*, 9(3), 602–611. 10.1080/13619469508581356

[cit0026] McLeod, J. (1966). Prediction of childhood dyslexia. *Bulletin of the Orton Society*, 16(1), 14–23. 10.1007/BF02928409

[cit0027] Miles, T. (1967). In defence of the concept of dyslexia. In J. Downing & A. L. Brown (Eds.), *The second international reading symposium*, 242-60. Cassell.

[cit0028] Miles, T. (2006). *Fifty years in Dyslexia research*. John Wiley.

[cit0029] Ministry of Education. (1946). *Special educational treatment*. HMSO.

[cit0030] Naidoo, S. (1972), *Specific Dyslexia: The reason report of the ICAA word blind centre for Dyslexic children* Pitman.

[cit0031] Prescott, H. M., Rauh, J. L., & Masland, R. P. (1996). In memorium: James Roswell Gallagher, MD, 1903–1995. *Journal of Adolescent Health*, 18(1), 2–3. 10.1016/1054-139X(95)00305-C

[cit0032] Rackham, K. (1978). *Invalid children’s aid association. The first ninety years*. ICAA.

[cit0033] Reid, J. F. (1968). Dyslexia: A problem of communication. *Educational Research*, 10(2), 126–133. 10.1080/0013188680100205

[cit0034] Rowland, T., & Hatch, G. (2007). Learning to Teach? The assistant lecturer in Colleges of Education, 1966–75. *History of Education*, 36(1), 65–88. 10.1080/00467600600909926

[cit0035] Rutter, M., Yule, W., Tizard, J., & Graham, P., 1968. ‘Severe reading retardation: Its relationship to maladjustment, epilepsy and neurological disorders’, in Association for Special Education, *What is special education? The proceedings of the 1st international conference (28th Biennial conference) of the association for special education, 25th-28th July 1968*, London: Association for Special Education.

[cit0036] Steedman, C. (1985). “The mother made conscious”: The historical development of a primary school pedagogy’. *History Workshop Journal*, 20(1), 149–163. 10.1093/hwj/20.1.149

[cit0037] Stein, Z., & Susser, M. (1960). Families of dull children: Part III – Social selection by family type. *Journal of Mental Science*, 106(445), 1304–1310. 10.1192/bjp.106.445.1304

[cit0038] Stewart, J. (2013). *Child guidance in Britain, 1918–1955: The dangerous age of childhood*. Pickering and Chatto.

[cit0039] Thom, D. (1992). Wishes, anxieties, play and gestures: Child guidance in interwar England. In R. Cooter (Ed.), *In the name of the child: Health and welfare*, 200-219. Routledge.

[cit0040] Thomas, J. B. (2007). Psychology of education in the UK: Development in the 1960s. *Educational Studies*, 33(1), 53–63. 10.1080/03055690600948182

[cit0041] Thomson, M. (2013). *Lost freedom: The landscape of the child and british post-war settlement*. *Times*, 4 October 1984: 16. Oxford University Press.

[cit0042] Tizard, J. (1972), *Children with specific reading difficulties: Report of the advisory committee on handicapped children* HMSO.

[cit0043] Vernon, M. (1966). Research on backwardness in reading. In J. Downing (Ed.), *The first international reading symposium. Oxford 1964* (pp. 148–159). Cassell.

[cit0044] White Franklin, A. (ed.). (1962). *Word-blindness or specific developmental Dyslexia: Proceedings of a conference called by the invalid children’s association, 12 April 1962*. Pitman.

[cit0045] White Franklin, A. (ed.) (1965), Children with communication problems : proceedings of a conference called by ICAA(the Invalid Children’s Aid Association), 7 April 1964 and held in the Medical College of St Bartholomew’s Hospital, London EC1. Pitman.

[cit0046] White Franklin, A., & Naidoo, S. (1970). *Assessment and treatment of Dyslexic children: Lectures given at a training course organized by the invalid children’s aid association word blind centre*. Pitman.

[cit0047] Wooldridge, A. (1994). *Measuring the mind: Education and psychology in England, ca. 1866 – ca. 1990*. Cambridge University Press.

[cit0048] Yule, W., & Rutter, M. (1970). Neurological aspects of intellectual retardation and specific reading retardation. In M. Rutter, J. Tizard, & K. Whitmore (Eds.), *Education, health and behaviour* (pp. 54–74). Longman.

[cit0049] Zangwill, O. (1960). *Cerebral dominance and its relation to psychological function*. Oliver and Boyd.

